# Role of urinary leukocytes in the risk stratification of prostate cancer using nonlinear stacking learning strategy: a bi-cohort diagnostic study

**DOI:** 10.3389/fonc.2026.1762494

**Published:** 2026-03-02

**Authors:** Shou Xia, Zhenchun Ran, Mengzhe Cheng, Hao Zhu, Chunguang Yang, Xinglong Wu

**Affiliations:** 1 Xianning Polytechnic, Xianning, China; 2 School of Computer Science and Engineering, Hubei Key Laboratory of Intelligent Robot, Wuhan Institute of Technology, Wuhan, China; 3 Aerospace Nanhu Electronic Information Technology Co., Ltd., Jingzhou, China; 4 Department of Urology, Tongji Hospital, Tongji Medical College, Huazhong University of Science and Technology, Wuhan, China; 5 Xinjiang Key Laboratory of Artificial Intelligence Assisted Imaging Diagnosis, Kashi, China

**Keywords:** prostate cancer, prostate-specific antigen, risk stratification, stacking learning, urinary leukocytes

## Abstract

**Objective:**

This study aimed to develop a non-linear stacking ensemble learning framework to evaluate the incremental diagnostic contribution of urinary leukocytes (UL) in prostate cancer (PCa) risk stratification, with a primary focus on predictive performance and clinical utility.

**Methods:**

We retrospectively included 492 men with elevated PSA levels from Tongji Hospital (n = 415) and Xiangyang Central Hospital (n = 77). All patients underwent transrectal ultrasound-guided prostate biopsy and were classified into low-, intermediate-, and high-risk PCa according to the Gleason score. Clinical variables, including age, BMI, tPSA, f/tPSA, p2PSA, PHI, PHID, and PSAD, were collected and standardized. Feature selection was performed using least absolute shrinkage and selection operator (LASSO) regression combined with bootstrap-based stability analysis. Based on the selected features, we constructed a non-linear stacking ensemble comprising decision tree, logistic regression, support vector machine (SVM), k-nearest neighbors (KNN), and gradient boosting as base learners. Three-class risk stratification models were trained under two scenarios: with and without incorporation of UL. Model performance was evaluated using the area under the receiver operating characteristic curve (AUC), macro-F1 score, and the DeLong test. Calibration curves and decision curve analysis (DCA) were applied to quantify the incremental net clinical benefit associated with UL. Predefined subgroup analyses across PSA strata (<10, 10–20, >20 ng/mL) were conducted to examine the context-dependent contribution of UL.

**Results:**

In the baseline setting without UL, the non-linear stacking model achieved AUCs of 0.962 and 0.928 in the internal and external cohorts, respectively, indicating robust discriminative performance. After incorporating UL, several base learners—particularly decision tree, KNN, and gradient boosting—demonstrated center-specific AUC improvements ranging from 0.003 to 0.02 (p < 0.05), accompanied by consistently increased net clinical benefit on DCA. Subgroup analyses showed that the incremental value of UL was most evident in patients with intermediate PSA levels (4–10 ng/mL) and in those with clinical features suggestive of benign prostatic hyperplasia. *Post hoc* SHapley Additive exPlanations (SHAP) analyses performed on a representative base learner indicated that UL exerted a modest but directionally consistent influence on high-risk predictions, complementing established PSA-derived indices rather than acting as a dominant independent driver.

**Conclusion:**

Within a stacking ensemble–based risk stratification framework primarily optimized for predictive performance, urinary leukocytes provide a clinically meaningful auxiliary signal that improves discrimination and net benefit in specific PSA-defined subgroups. These findings support the use of UL as a complementary inflammation-related marker in PCa risk assessment, while interpretability is best understood at the level of base learners and original clinical features rather than the full ensemble model.

## 1 Introduction

Prostate cancer (PCa) remains one of the most prevalent malignancies among men worldwide and continues to impose a substantial and enduring burden on public health systems (1). Accurate risk stratification constitutes a pivotal component of contemporary PCa management. Patients with indolent or low-risk disease may be safely managed with active surveillance to avoid overtreatment, whereas those with intermediate- or high-risk disease require timely definitive therapy to mitigate the risks of metastasis and disease-specific mortality. Current clinical guidelines commonly stratify patients into low-, intermediate-, and high-risk groups—or more granular subcategories—based on prostate-specific antigen (PSA) levels, Gleason score/ISUP grade, and clinical stage. Extensive evidence indicates that such multidimensional risk systems are strongly associated with tumor grade, therapeutic decision pathways, and long-term outcomes, helping reduce unnecessary biopsies and overtreatment while maintaining the detection rate of clinically significant disease (2–4). In contemporary clinical practice, prostate cancer risk stratification integrates histopathological and imaging-based assessments. The Gleason score, obtained from prostate biopsy, defines tumor aggressiveness and definitive risk categories, whereas PI-RADS provides an MRI-based estimate of lesion suspicion prior to biopsy and guides biopsy indication and targeting.

Despite their clinical utility, conventional risk stratification models face two persistent challenges in real-world settings. First, PSA and its derivative indices lack sufficient specificity; elevations may arise from benign prostatic hyperplasia (BPH), prostatitis, or subclinical inflammation. Second, many existing stratification approaches rely on linear or near-linear assumptions, limiting their ability to capture the nonlinear and interactive relationships among heterogeneous clinical variables, thereby constraining the accuracy of personalized decision-making.

Among various liquid biomarkers, urine has clear advantages—noninvasiveness, ease of repeated collection, and anatomical proximity to the prostate. Urine-based assays such as PCA3,TMPRSS2:ERG, and ExoDx Prostate have been successfully developed, building on foundational work by Fujita et al. (5). In contrast, routine urinalysis parameters—particularly urinary leukocytes (UL)—have long been overlooked in PCa risk stratification. UL are typically interpreted as markers of urinary tract infection or sterile inflammation (4). Elevated UL may influence PSA interpretation and prostate volume assessments; however, prior studies have largely regarded UL as a confounder rather than a potentially informative feature (6). While some evidence suggests an association between sterile pyuria and aberrant PSA elevation (5), its relationship with biopsy positivity has been inconsistent, implying that inflammatory processes may introduce systematic bias into PSA-based diagnostic reasoning (7). Within the context of precision medicine, a natural yet unresolved question emerges: if UL are no longer treated as noise but are integrated alongside PSA-derived markers within data-driven models, can they contribute incremental information to enhance PCa risk stratification?

With the rapid adoption of machine learning and deep learning in urologic oncology, numerous models—including random forests, support vector machines, gradient boosting machines, neural networks, and radiomics-based classifiers—have been proposed for early PCa detection and risk prediction (8). Many demonstrate superior discriminative performance compared with traditional regression models, and decision curve analyses often show improved net clinical benefit (2). However, the vast majority of existing studies focus on “canonical” predictors such as PSA, PHI, PHID, PSAD, and MRI-derived features; routine urinalysis markers have been sparsely explored. To date, no study has explicitly incorporated urinary leukocytes into a multiclass PCa risk stratification model or rigorously assessed their incremental diagnostic value using nonlinear ensemble learning frameworks in multi-center cohorts (9).

To fill this methodological and clinical gap, we proposed a nonlinear ensemble learning strategy incorporating urinary leukocytes as a candidate predictive feature for three-class PCa risk stratification (10). Leveraging real-world data from 492 patients across Tongji Hospital and Xiangyang Central Hospital, we applied LASSO with bootstrap stability selection to identify robust predictors and subsequently constructed a nonlinear stacking framework integrating gradient boosting machines, support vector machines, logistic regression, and k-nearest neighbors. Two parallel models—without UL and with UL—were developed. Using micro-ROC AUC, macro-F1, calibration curves, DeLong tests, and decision curve analysis, we quantified the incremental value of UL and assessed its consistency across PSA subgroups. In addition, SHAP-based model interpretability was employed to characterize nonlinear interactions between UL and key markers such as PSAD and PHI, and to delineate patient-level decision contributions (11).

This study features several methodological and clinical innovations: (i) to our knowledge, it is the first multicenter analysis to systematically evaluate the additive diagnostic value of routine urinalysis indicators—specifically urinary leukocytes—in three-class PCa risk stratification; (ii) the nonlinear ensemble learning and multi-metric decision analytic framework better approximates real-world decision boundaries than traditional linear models; and (iii) the interpretability analyses provide mechanistic insights into whether UL should be retained as a cost-effective and clinically meaningful feature within PCa risk models. Collectively, this work offers data-driven evidence to support whether and how UL may be judiciously integrated into contemporary PCa risk assessment workflows.

## 2 Participants and methods

This retrospective study was approved by the Ethics Committee of Tongji Hospital, Tongji Medical College, Huazhong University of Science and Technology (approval No. TJ-IRB202303129), with a waiver of written informed consent. All data were de-identified prior to analysis and used exclusively for research purposes.

### 2.1 Participants

#### 2.1.1 Patient sources and eligibility criteria

A total of 492 patients were included in this study, comprising 415 cases from Tongji Hospital and 77 cases from Xiangyang Central Hospital. The internal cohort consisted of 415 men with elevated PSA levels who presented to Tongji Hospital between December 2022 and March 2024. The external cohort included men with elevated PSA levels who presented to Xiangyang Central Hospital between April and May 2024. Clinical information and histopathological findings were collected for all participants. Inclusion criteria were as follows: (1) availability of accurate clinical information sufficient for calculating PSA-derived parameters; (2) prostate MRI performed prior to biopsy; (3) MRI quality adequate for Prostate Imaging–Reporting and Data System (PI-RADS) scoring. Exclusion criteria were: missing key clinical variables (e.g. PSA level, pathological Gleason score) that could not be retrieved from the medical records, and absence of urinary leukocyte measurements.

#### 2.1.2 Risk stratification

Pathological Gleason score was used to define three risk categories (12), which served as the three-class labels (low/intermediate/high) for the machine learning models: low-risk, Gleason score ≤ 6; intermediate-risk, Gleason score = 7; and high-risk, Gleason score ≥ 8.

Training and validation cohorts: Data from Tongji Hospital were used for model development and internal validation. To preserve the overall distribution and the proportion of risk categories, the Tongji cohort was randomly split into a training set and an internal test set at a 7:3 ratio using stratified sampling. Data from Xiangyang Central Hospital were not used for feature selection or model training and were reserved as an independent external validation cohort to assess model generalizability and stability.

#### 2.1.3 Urinary leukocytes assessment

Urinary leukocytes were obtained from routine pre-biopsy urinalysis performed on fresh midstream urine samples as part of standard clinical work-up. Urinalysis was conducted using automated urine analyzers routinely deployed in each participating center, following manufacturers’ standard operating procedures.

In the present study, UL was treated as a binary variable and defined as positive when leukocyte esterase was reported as ≥ trace/”+” on dipstick urinalysis, and negative otherwise. This threshold corresponds to standard clinical reporting practice and reflects the presence of urinary inflammatory activity rather than a quantitative leukocyte burden.

We did not explore alternative UL cut-offs in the primary analysis, as semi-quantitative dipstick grading (negative, trace, +, ++, +++) is not fully standardized across analyzers and centers. Instead, UL was intentionally modeled as a coarse binary indicator to ensure robustness and clinical generalizability across institutions.

### 2.2 Clinical characteristics

The clinical and pathological characteristics of patients in the training, internal test and external validation cohorts are summarized in **Table 1**.

**Table 1:** Clinical and pathological characteristics of patients in the retrospective training and test cohorts.

Characteristics	Retrospective training and test sets	Retrospective training and test sets	P-Values
number of patients	415	77	
AGE(years)	66.00 (60.00–72.00)	68.00 (61.00–72.00)	0.2282
BMI(kg/m²)	23.66 (21.95–25.63)	23.70 (21.80–25.80)	0.9703
tPSA(ng/ml) (ng/ml)	9.37 (6.35–14.67)	10.28 (6.42–15.63)	0.7372
fPSA(ng/ml)	1.48 (0.98–2.25)	1.19 (0.85–1.77)	0.0215*
fPSA/tPSA(ng/ml)	15.75 (10.89–22.23)	12.61 (9.10–17.05)	<0.001*
p2PSA(ng/ml)	19.81 (12.82–31.77)	18.24 (12.23–33.28)	0.8772
PHI	41.70 (28.00–65.45)	49.60 (30.10–81.10)	0.0562
PSAD(ng)	0.20 (0.12–0.36)	0.19 (0.13–0.40)	0.9708
PHID	0.86 (0.47–1.93)	0.92 (0.52–2.47)	0.2677
Neutrophils(10^^9^/L)	3.41 (2.70–4.22)	3.90 (3.18–5.32)	0.0013*
Lymphocytes(10^^9^/L)	1.61 (1.29–1.92)	1.48 (1.08–1.81)	0.0549
NLR(10^^9^/L)	2.17 (1.68–2.91)	2.87 (1.76–4.07)	<0.001*
Volume(mL)	46.44 (30.51–66.04)	44.60 (31.20–72.20)	0.8645
Gleason Score, n (%)			
Benign	270 (65.1%)	47 (61.0%)	
Intermediate	105 (25.3%)	20 (26.0%)	
High	40 (9.6%)	10 (13.0%)	
Smoking history, n (%)			0.1546
0	302 (72.8%)	62 (80.5%)	
1	113 (27.2%)	15 (19.5%)	
urinary leukocytes, n (%)			0.0612
0	299 (72.6%)	65 (84.4%)	
1	113 (27.4%)	12 (15.6%)	
PIRADS, n (%)			0.7457
1	8 (1.9%)	0 (0.0%)	
2	86 (20.7%)	14 (18.2%)	
3	157 (37.8%)	30 (39.0%)	
4	83 (20.0%)	16 (20.8%)	
5	81 (19.5%)	17 (22.1%)	

*indicates a statistically significant difference.

### 2.3 Study pipeline

#### 2.3.1 Overall workflow and modeling framework

**Figure 1:**
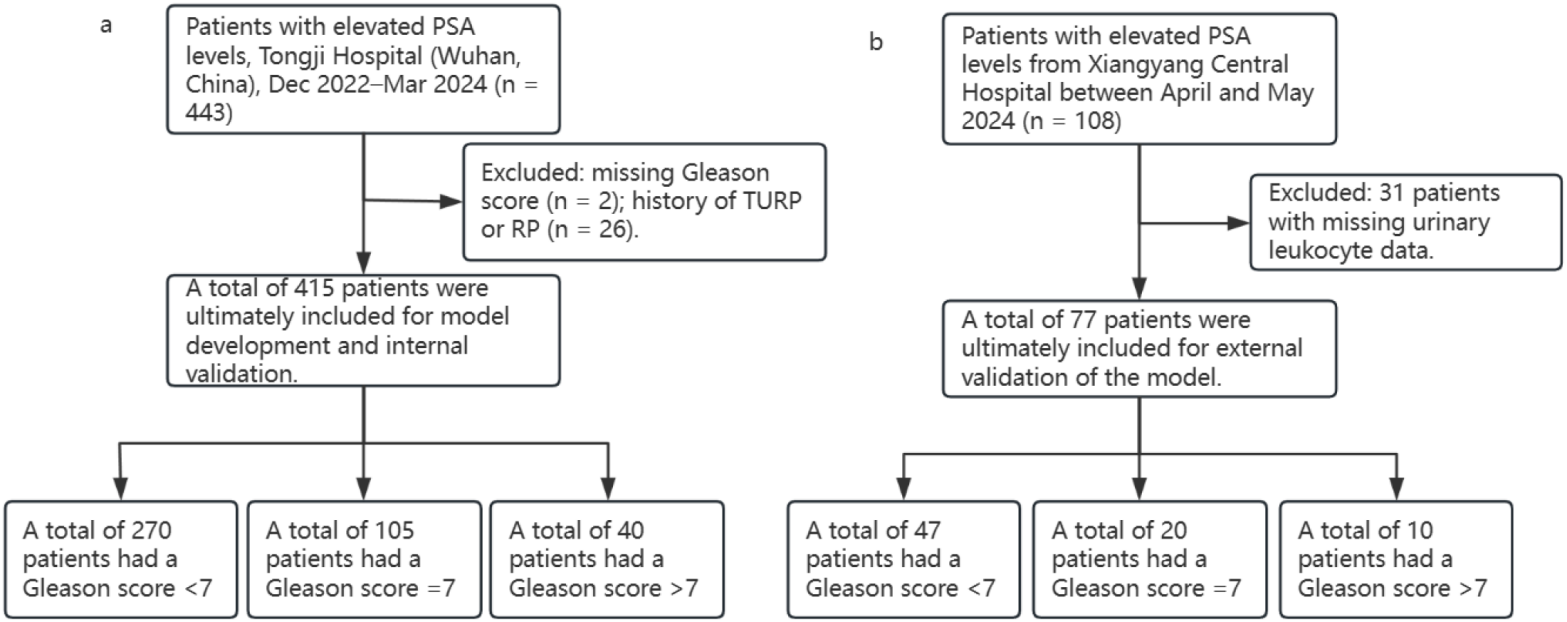
Flowchart of data screening and cohort allocation. **(a)** Internal dataset; **(b)** External validation dataset.

The overall study workflow and modeling pipeline are schematically illustrated in **Figure 1**.

#### 2.3.2 Feature engineering and variable selection

To fully capture potential nonlinear relationships between prostate-related indices and tumor risk, while controlling the risk of overfitting in a high-dimensional feature space, we performed systematic feature engineering and regularized variable selection prior to model development.

Based on prior literature and clinical expertise, we first identified a predefined set of baseline clinical and biochemical variables, including age, body mass index (BMI), total PSA (tPSA), free-to-total PSA ratio (f/tPSA), p2PSA, Prostate Health Index (PHI), PHI density (PHID), PSA density (PSAD), PI-RADS score, prostate volume, and Lymphocytes cell counts. PHI and p2PSA have been shown in multicenter cohorts and systematic reviews to significantly improve discrimination of clinically significant PCa beyond conventional tPSA, with superior AUC and decision-curve performance compared with PSA-only models (13). PSAD and PHID have been repeatedly validated as important parameters for identifying high-risk lesions and reducing unnecessary biopsies, whereas PI-RADS v2/v2.1 has become a standardized tool for mpMRI-based assessment of suspicious lesions and their correspondence with Gleason grading (14). Collectively, these data support using the above variables as the core feature set for model construction and extension in the present study.

**Figure 2:**
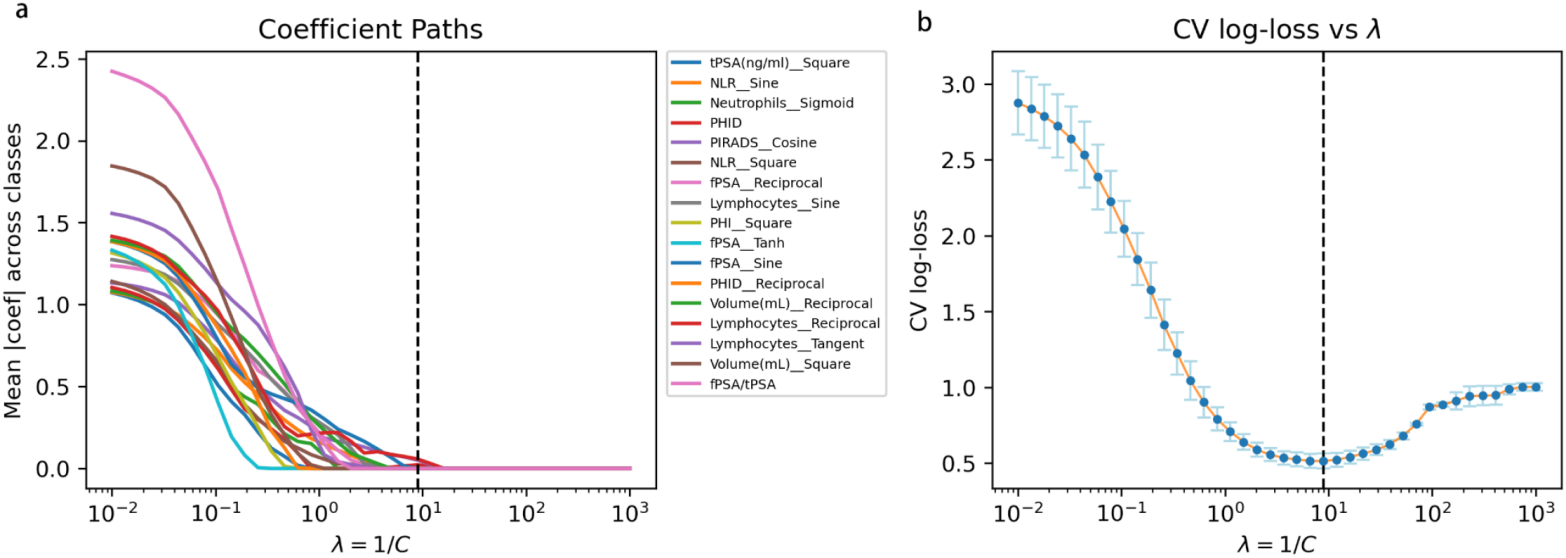
Nonlinear feature selection using multiclass LASSO. **(a)** coefficient paths of candidate nonlinear features as a function of λ (= 1/C). As λ increases, model sparsity increases and weak predictors are progressively eliminated. **(b)** 5-fold cross-validated multiclass log-loss as a function of λ. The dashed line indicates the selected λ, which lies within the near-optimal performance region while yielding a sparse feature set comprising 17 stable predictors.

Given that these indices typically exhibit skewed distributions, threshold effects and saturation effects, we predefined, using only the internal training set, a library of statistically plausible nonlinear transformations for each candidate variable (15). These transformations included, but were not limited to, logarithm (log), square root (sqrt), reciprocal, square, hyperbolic tangent (tanh), sigmoid, tangent (tan), and simple sinusoidal transformations (sin/cos). Importantly, these transformations were not intended to represent explicit physiological mechanisms for each variable; rather, they serve as generic nonlinear basis functions that allow the models to capture complex, potentially non-monotonic relationships in a data-driven manner. All transformations were specified *a priori*, and the same rules were applied uniformly across variables to generate an expanded feature set, thereby constructing a rich yet interpretable candidate feature space rather than performing outcome-driven, *post hoc* feature engineering (**Figure 2a**).

After nonlinear expansion, we applied L1-regularized multinomial logistic regression (least absolute shrinkage and selection operator, LASSO) for feature selection (16). By introducing an L1 penalty term into the loss function, LASSO shrinks coefficients of non-informative or highly redundant features exactly to zero, thus achieving parameter estimation and dimensionality reduction within a single framework. This approach is particularly suited to medical datasets in which the number of features approaches or exceeds the sample size (17). The regularization parameter λ was chosen on the internal training set via K-fold cross-validation, with the average multinomial log-loss used as the optimization criterion. As illustrated in **Figure 2b**, the cross-validated log-loss reached its minimum at an optimal λ (dashed vertical line), reflecting the best trade-off between predictive accuracy and model sparsity. This data-driven selection ensured that only the most informative nonlinear features were retained while reducing the risk of overfitting.

To assess the stability of variable selection, we employed a resampling strategy (bootstrap or repeated cross-validation) within the training set, refitting the LASSO model in each resampled dataset (18) and recording the selection frequency of every candidate feature. Only features that were consistently selected in the majority of resampling iterations were retained as the final modeling feature set. These features were then used to train the subsequent nonlinear machine learning models (gradient boosting, support vector machine, logistic regression, k-nearest neighbors, etc.) under two scenarios: without UL and with UL. This pipeline ensured reproducible and interpretable feature engineering while minimizing the influence of stochastic noise on model construction.

#### 2.3.3 Ensemble learning strategy

We first trained and evaluated more than ten commonly used machine learning algorithms on the training cohort using 5-fold cross-validation, including logistic regression (Logistic Regression), support vector machine (SVM), k-nearest neighbors (kNN), decision tree (Decision Tree), random forest (Random Forest, RF), gradient boosting decision tree (Gradient Boosting), extreme gradient boosting (XGBoost), Light Gradient Boosting Machine (LightGBM), naïve Bayes (Naive Bayes) and multilayer perceptron (MLP). Hyperparameters for each model were tuned by grid search, an exhaustive procedure that iterates over all predefined combinations within a hyperparameter grid, trains and evaluates each configuration, and selects the one achieving the best cross-validated performance on the validation folds (19).

We then designed an ensemble learning strategy tailored for PCa risk stratification and multi-class classification. The five best-performing base learners were selected according to their cross-validated performance. For these five models, hyperparameters were further optimized using grid search combined with 5-fold cross-validation, and the models were trained and fine-tuned on the retrospective cohorts, yielding a set of high-performing and mutually complementary base classifiers that underpin the subsequent ensemble schemes. Two ensemble approaches were constructed and compared: voting and stacking (20, 21). In the voting ensemble, we implemented an equal-weight soft-voting scheme, in which the class probabilities output by the five probability-calibrated base models were averaged without weighting to obtain the final prediction (22). In the stacking ensemble, we adopted a two-layer architecture. The first layer consisted of the same five base models; using 5-fold cross-validation on the training set, we obtained out-of-fold class probability predictions for each model, and concatenated these multi-class probability vectors to form a new set of meta-features. The second layer employed L2-regularized logistic regression as the meta-learner (23), which learns an optimal linear combination of the base-model outputs without information leakage. This design allows automatic estimation of the contribution (weights) of each base learner and aims to further improve overall predictive performance. All ensembles were constructed and evaluated under two scenarios—without UL and with UL—to quantify the incremental value of urinary leukocytes for PCa risk stratification.

#### 2.3.4 Statistical analysis

Continuous variables (**Table 2**) are summarized as median and interquartile range (IQR) and were compared between groups using the Mann–Whitney U test. Categorical variables are presented as counts and percentages and were compared using the χ² test. All statistical tests were two-sided, and a P value < 0.05 was considered statistically significant.

**Table 2:** Performance of multiple models on the internal test set and external validation cohort.

Dataset	Model	AUC (95%CI)	Accuracy	MacroF1	MacroPPV	MacroNPV	Pos2_ Sensitivity	Pos2_ Specificity
***Internal***	Decision Tree	0.927 (0.898-0.954)	0.81	0.60	0.83	0.90	0.13	1
	Gradient Boosting	0.947 (0.912-0.976)	0.84	**0.70**	0.74	0.93	0.38	0.97
	KNN	0.936 (0.903-0.968)	0.83	0.69	0.74	0.92	0.38	0.97
	Logistic Regression	0.952 (0.934-0.970)	0.81	0.66	0.75	0.93	0.38	0.99
	SVM	0.949 (0.920-0.972)	0.81	0.67	0.71	0.92	**0.88**	0.88
	Stacking	**0.962 (0.936-0.983)**	**0.86**	0.69	**0.87**	**0.94**	0.25	**1**
	Voting	0.956 (0.930-0.978)	0.84	0.67	0.86	**0.94**	0.25	**1**
***External***	Decision Tree	0.925 (0.897-0.951)	0.77	0.61	0.80	0.88	0.20	**1**
	Gradient Boosting	0.920 (0.878-0.953)	0.75	0.64	0.70	0.88	0.50	0.97
	KNN	0.884 (0.839-0.923)	0.70	0.55	0.59	0.84	0.30	0.96
	Logistic Regression	0.913 (0.880-0.944)	**0.78**	**0.67**	0.73	**0.92**	0.60	0.97
	SVM	0.921 (0.882-0.956)	0.75	**0.67**	0.66	0.87	**0.80**	0.87
	Stacking	**0.928 (0.897-0.954)**	0.73	0.52	0.54	0.87	0.10	0.96
	Voting	0.927 (0.895-0.955)	0.75	0.58	0.68	0.89	0.20	0.99

(Pos2_* denotes performance metrics calculated with class 2 as the positive class; in this study, class 2 corresponds to the high-risk group. Bold values indicate the best-performing model for each metric within the corresponding dataset).

All statistical analyses and model development were performed in Python 3.11.11. Classical statistical tests and P value calculations were conducted using the SciPy library (two-sided tests with a significance level α = 0.05). Machine learning models were implemented with scikit-learn for model orchestration and pre-processing. For tree-based models, XGBoost (version 3.0.1) and LightGBM (version 4.6.0) were integrated through their respective scikit-learn compatible APIs. Specifically, XGBoost was used via the XGBClassifier from the xgboost library, and LightGBM was used via the LGBMClassifier from the lightgbm library. Other models, including RandomForest, DecisionTree, LogisticRegression, SVM, and KNN, were directly implemented using scikit-learn. Model interpretability analyses were carried out using the shap library (SHapley Additive exPlanations). Figures and graphical summaries were generated using matplotlib and seaborn. To enhance reproducibility, random seeds were fixed across all training and evaluation procedures.

Model performance was quantified using several metrics, including the area under the receiver operating characteristic curve (AUC), sensitivity, specificity and macro-averaged F1 score (macro-F1) (24). Sensitivity measures the ability of the model to correctly identify positive cases, whereas specificity measures its ability to correctly identify negative cases. The F1 score, defined as the harmonic mean of precision and recall, provides a composite measure that jointly accounts for false positives and false negatives and is particularly suitable for imbalanced multi-class classification (25).

## 3 Results

### 3.1 Overall performance evaluation

**Figure 3:**
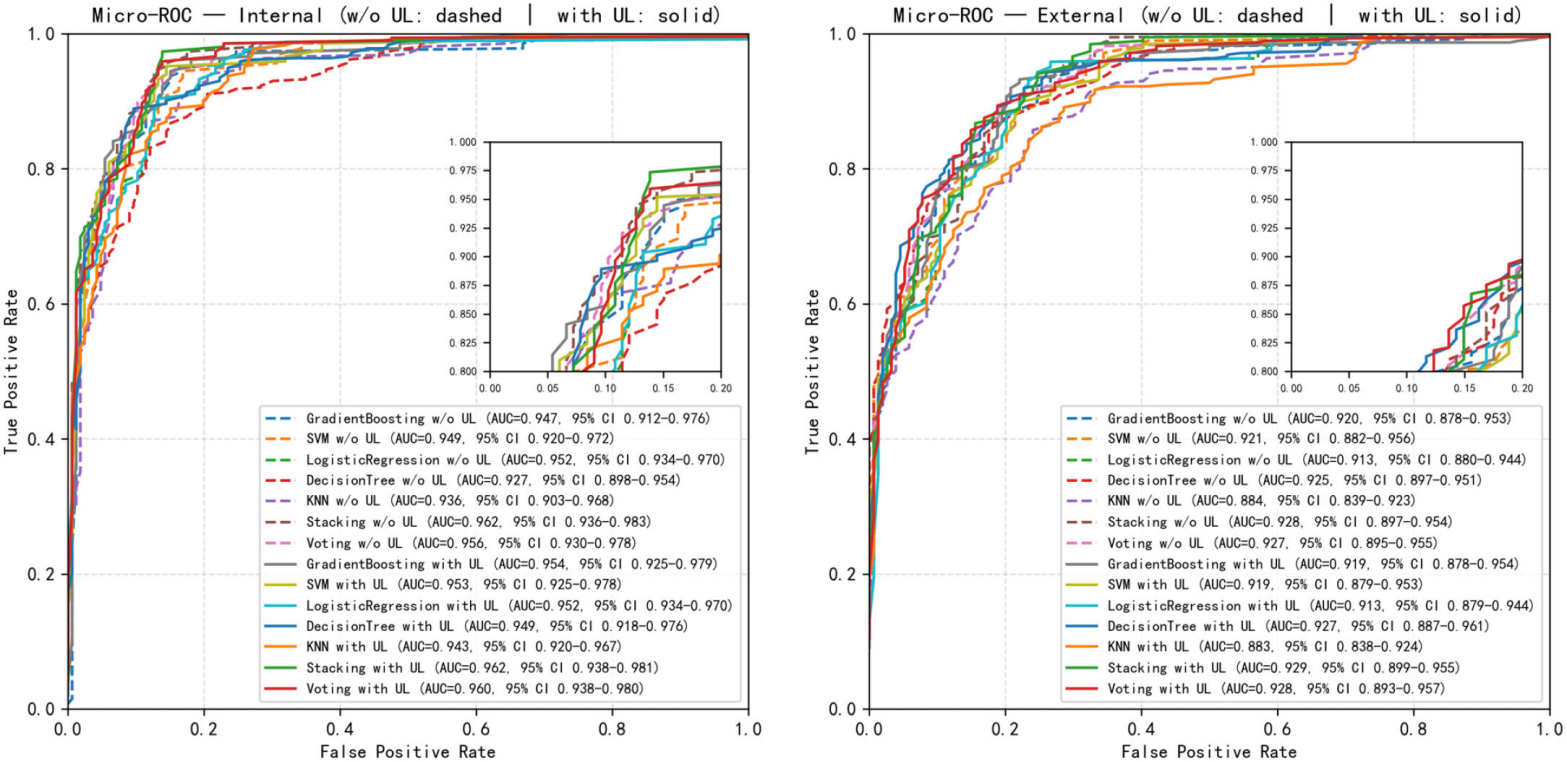
Micro-averaged ROC curves of multiple models in the internal test set and external validation cohort.

Among all candidate algorithms, the five best-performing base learners were gradient boosting, support vector machine (SVM), logistic regression, k-nearest neighbors (KNN) and decision tree. **Figure 3** depicts the micro-averaged ROC curves of these models, and the corresponding quantitative results are summarized in **Table 2**. At the single-model level, all classifiers already achieved high discriminative performance, with AUCs ranging from 0.927 to 0.952; logistic regression yielded the highest AUC of 0.952 (95% CI, 0.934–0.970). On top of this strong baseline, the ensemble strategies further improved overall performance: the stacking ensemble achieved an AUC of 0.962 (95% CI, 0.936–0.983), and the voting ensemble achieved an AUC of 0.956 (95% CI, 0.930–0.978), both outperforming any individual base learner. Stacking also showed slightly higher accuracy (0.86 vs. 0.84) and macro-averaged F1 score (0.69 vs. 0.67) compared with voting. Macro-averaged PPV (0.86–0.87) and macro-averaged NPV (both 0.94), together with specificities approaching 1.000, indicated good negative-class recognition and overall stability for both ensembles. When high-risk PCa (Pos2) was treated as the positive class, SVM as a single model achieved the highest sensitivity (0.88), whereas the stacking and voting ensembles exhibited lower sensitivity for Pos2 (0.25 at the default decision threshold), reflecting a more conservative decision profile that prioritizes reduction of false positives.

External validation in the independent cohort showed that all models maintained good discrimination, with AUCs ranging from 0.884 to 0.928. The stacking and voting ensembles again performed slightly better than most single models, with AUCs of 0.928 (95% CI, 0.897–0.954) and 0.927 (95% CI, 0.895–0.955), respectively, compared with, for example, decision tree (AUC = 0.925), logistic regression (AUC = 0.913) and SVM (AUC = 0.921). In the external cohort, voting showed a modest advantage in terms of overall accuracy (0.75) and specificity (0.99), whereas stacking provided a more balanced trade-off between sensitivity and specificity, and between PPV and NPV, across clinically relevant decision thresholds (**Table 2**; **Figure 3**).

Taken together, the ensemble learning strategies enhanced the overall discriminative ability and robustness of PCa risk stratification beyond what was achievable with individual models alone. Among them, stacking appears more suitable when the primary objective is to maximize predictive performance and net clinical benefit, whereas voting—characterized by a simpler structure and consistently high specificity and NPV—offers an attractive and operationally straightforward option for clinical implementation.

### 3.2 Diagnostic value of UL for PCa risk stratification

To systematically quantify the incremental diagnostic value conferred by UL, we evaluated both calibration and clinical decision-analytic performance before and after incorporating UL into the models. Calibration was assessed using the multiclass Brier score and stratified calibration plots to examine the agreement between predicted probabilities and observed risks, and to compare the extent of calibration improvement after adding UL. Clinical utility was evaluated using decision curve analysis (DCA), which estimates the net benefit across a range of threshold probabilities (PT). We further computed ΔNB and ΔTP/ΔFP per 100 patients to visually illustrate the impact of UL on key clinical decisions (e.g., whether to perform biopsy or escalate management).

### 3.3 Clinical applicability of UL for PCa risk stratification

**Figure 4:**
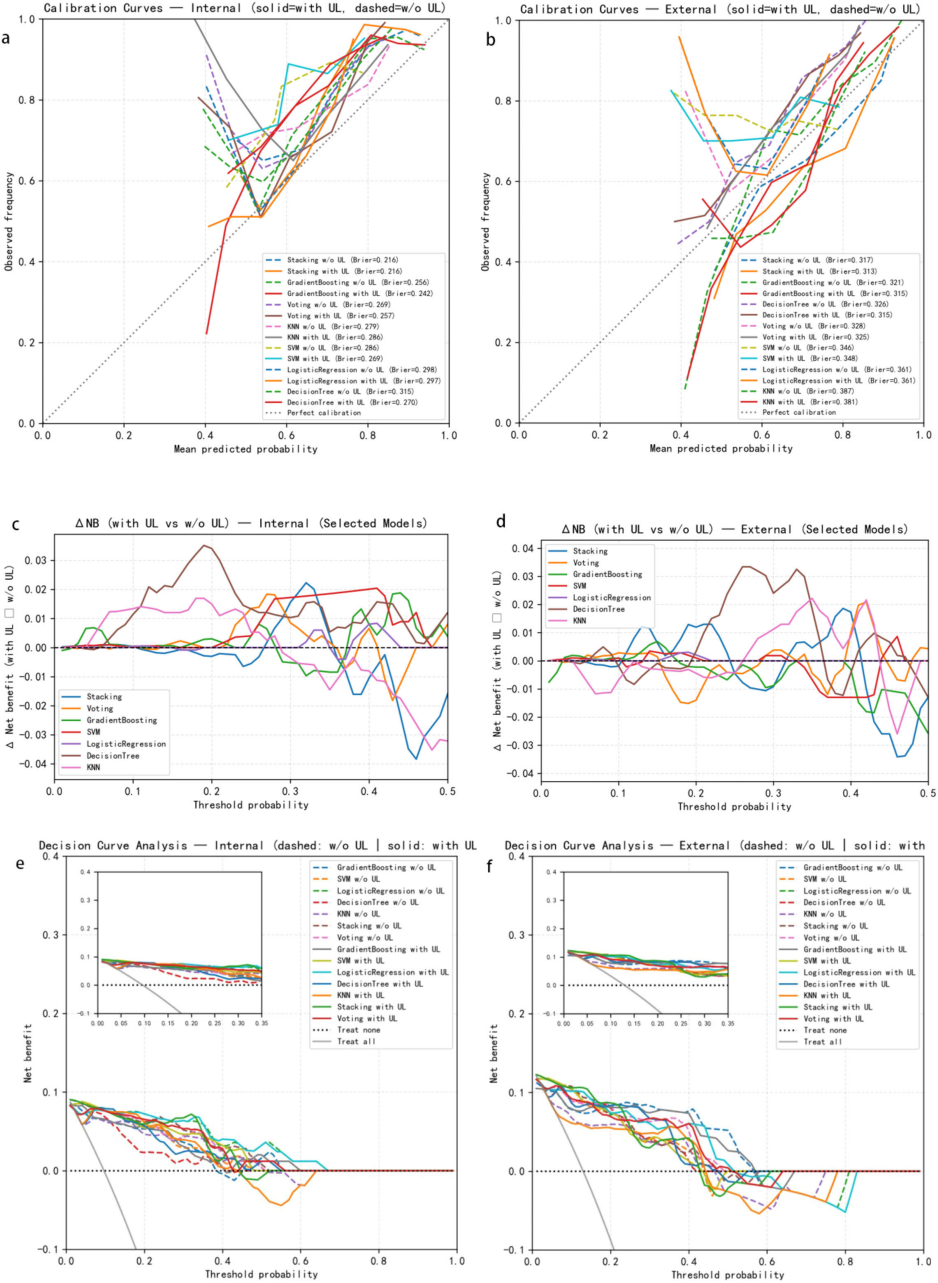
Discrimination, calibration, and clinical utility of models with and without urinary leukocytes (UL). **(a, b)** Calibration curves for the internal cohort **(a)** and external cohort **(b)**. Solid lines indicate models with UL; dashed lines indicate models without UL. **(c, d)** Differences in net benefit (ΔNB) between models with and without UL in the internal **(c)** and external **(d)** cohorts. **(e, f)** Decision curve analysis showing net benefit across a range of threshold probabilities for models with and without UL.

As shown in **Figures 4a–f**, incorporation of UL led to a consistent improvement in calibration performance in both the internal and external cohorts. In the internal dataset, the multiclass Brier score decreased after adding UL (e.g. from 0.216 to 0.210 for stacking, from 0.257 to 0.247 for voting, and from 0.256 to 0.242 for gradient boosting), indicating better agreement between predicted probabilities and observed risks. Stratified calibration curves further demonstrated that the with UL models (solid lines) were generally closer to the 45° identity line than their without UL counterparts (dashed lines), suggesting a reduction in over- or underestimation, particularly in the high-risk probability range.

Decision curve analysis (DCA) provided complementary evidence at the clinical decision level. Within clinically relevant threshold probability ranges (PT ≈ 0.05–0.30), the with UL curves for all models lay above both the without UL curves and the “treat-all”/”treat-none” strategies. Among them, the stacking and voting ensembles yielded the highest net benefit, with peak ΔNB values of approximately 0.03–0.04 in the internal cohort, corresponding to an increase of about 2–4 correctly identified high-risk patients per 100 individuals (ΔTP ≈ 2–4/100 patients), without a substantial increase in false positives. In the external validation cohort, the absolute magnitude of ΔNB was somewhat smaller (on the order of 0.01–0.02) and more variable, but the overall pattern remained consistent, with UL models providing higher net benefit across a broad range of decision thresholds.

Taken together, these findings indicate that incorporating UL improves probability calibration and net clinical benefit for PCa risk stratification, while not materially increasing the risk of false positives. The incremental gain is stable across different PSA ranges and is particularly pronounced for ensemble models such as stacking and voting, supporting the role of UL as an independent, quantifiable biomarker with added diagnostic value in PCa risk prediction.

### 3.4 PSA-based subgroup analysis

**Figure 5:**
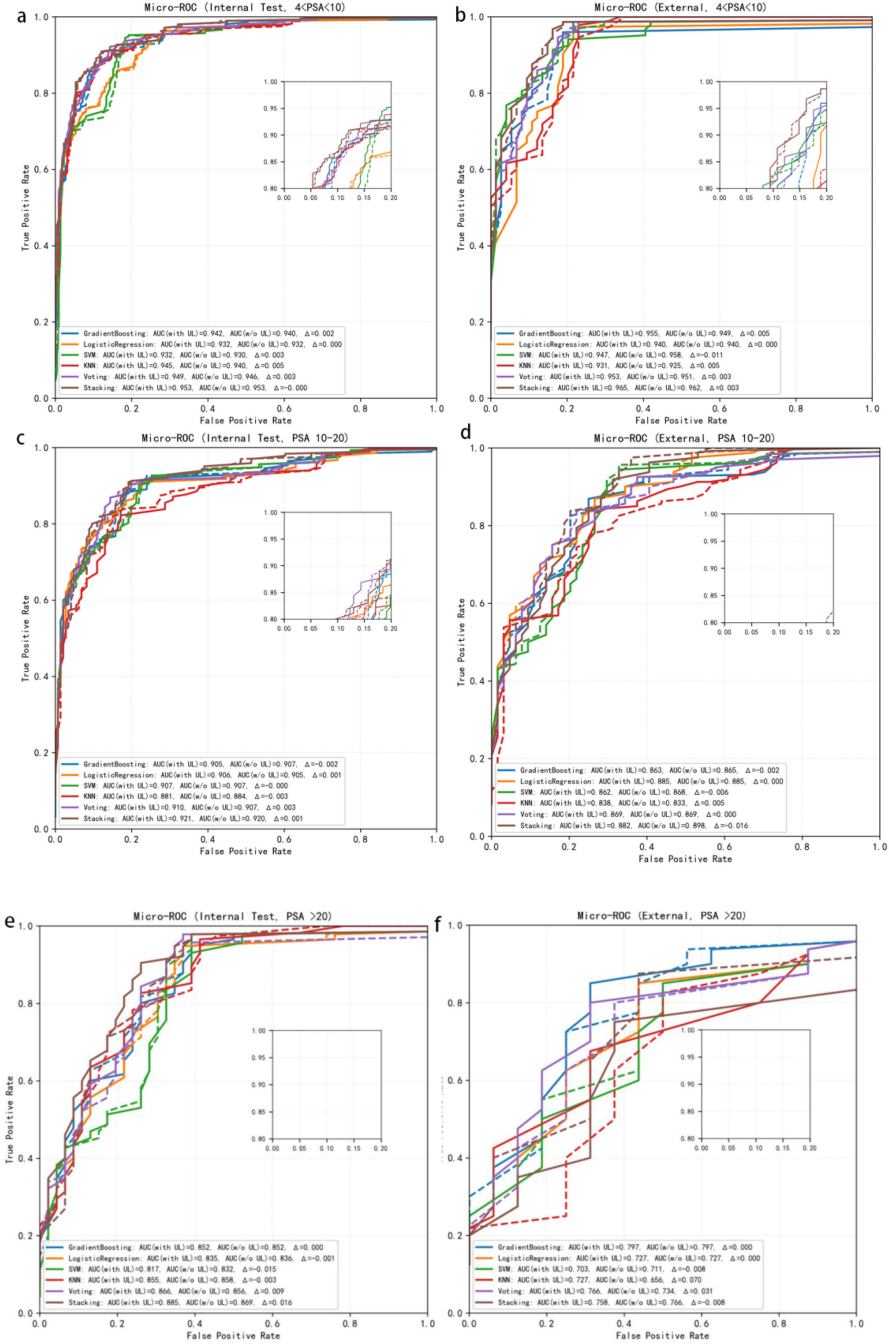
Micro-ROC curves of models with and without urinary leukocytes (UL) across baseline PSA subgroups. **(a, b)** Results for PSA 4–10 ng/mL; **(c, d)** results for PSA 10–20 ng/mL; **(e, f)** results for PSA >20 ng/mL. Solid lines denote models incorporating UL, whereas dashed lines denote the corresponding models without UL.

To investigate whether the incremental value of UL varies across different levels of tumor burden, we performed a pre-specified subgroup analysis stratified by baseline PSA. In the internal test set (**Figures 5a, c, e**), all models maintained high discriminative ability within the PSA 4–10 and 10–20 ng/mL strata, with micro-averaged AUCs clustering around 0.93. Upon incorporation of UL, none of the models showed performance deterioration in any subgroup; instead, the ROC curves shifted slightly towards the upper left. In the PSA 4–10 ng/mL subgroup, the absolute micro-AUC gains attributable to UL were modest but directionally consistent across model families—tree-based, linear and ensemble learners—with ΔAUC values largely confined to a narrow range of 0.002–0.005, suggesting a small yet robust incremental signal at low-to-intermediate PSA levels. In the PSA 10–20 ng/mL subgroup, the magnitude of AUC improvement was further attenuated, and ΔAUC varied slightly across algorithms, but changes remained predominantly small and positive with no evidence of systematic degradation. For the PSA >20 ng/mL subgroup, baseline micro-AUCs ranged from approximately 0.82 to 0.89; after adding UL, most models (particularly SVM, KNN and the Voting/Stacking ensembles) exhibited AUC increases of about 0.01–0.02, whereas Gradient Boosting and Logistic Regression were essentially unchanged, indicating that the contribution of UL in the high-PSA setting is more uncertain but, overall, unlikely to behave as pure noise.

Findings in the external validation cohort were broadly concordant with those of the internal cohort, albeit with greater variability (**Figures 5b, d, f**). Among patients with PSA 4–10 ng/mL, micro-AUCs for all models were consistently slightly higher after incorporating UL than for their respective baselines, with ΔAUC pointing in the same (favorable) direction, supporting the reproducibility of UL-related gains in the low-PSA range in an independent dataset. In the PSA 10–20 ng/mL subgroup, AUC differences largely fluctuated around zero, with only minor positive or negative deviations in individual models, indicating that the overall benefit approaches a marginal effect. In the PSA >20 ng/mL subgroup, where sample size was smaller and class imbalance more pronounced, ΔAUC values across algorithms included small positive shifts as well as values close to zero or slightly negative, and the ROC curves were visibly stepwise, underscoring limited estimation precision and an exploratory rather than definitive nature of the evidence in this range.

Taken together, UL did not compromise the discriminative performance of any model across PSA strata and provided a small but consistent incremental discriminative benefit within the PSA 4–20 ng/mL interval, with the clearest and most reproducible gains observed in the PSA 4–10 ng/mL low-to-intermediate PSA population. For patients with PSA >20 ng/mL, the direction of UL’s effect appears broadly non-negative in the current data, but given sample size constraints and unstable ROC estimation, conclusions in this high-PSA subgroup should be interpreted cautiously and confirmed in larger, multi-center cohorts.

### 3.5 SHAP-based interpretability analysis

SHAP analyses were conducted using the TreeExplainer, which is optimized for tree-based models, applied to the gradient boosting classifier. The internal training cohort was used as the background reference distribution. SHAP values were computed in a one-vs-rest framework for the high-risk class (class 2), such that positive values indicate increased predicted probability of high-risk PCa and negative values indicate a down-weighting effect. This formulation facilitates direct interpretation of feature contributions to clinically significant disease risk.

**Figure 6:**
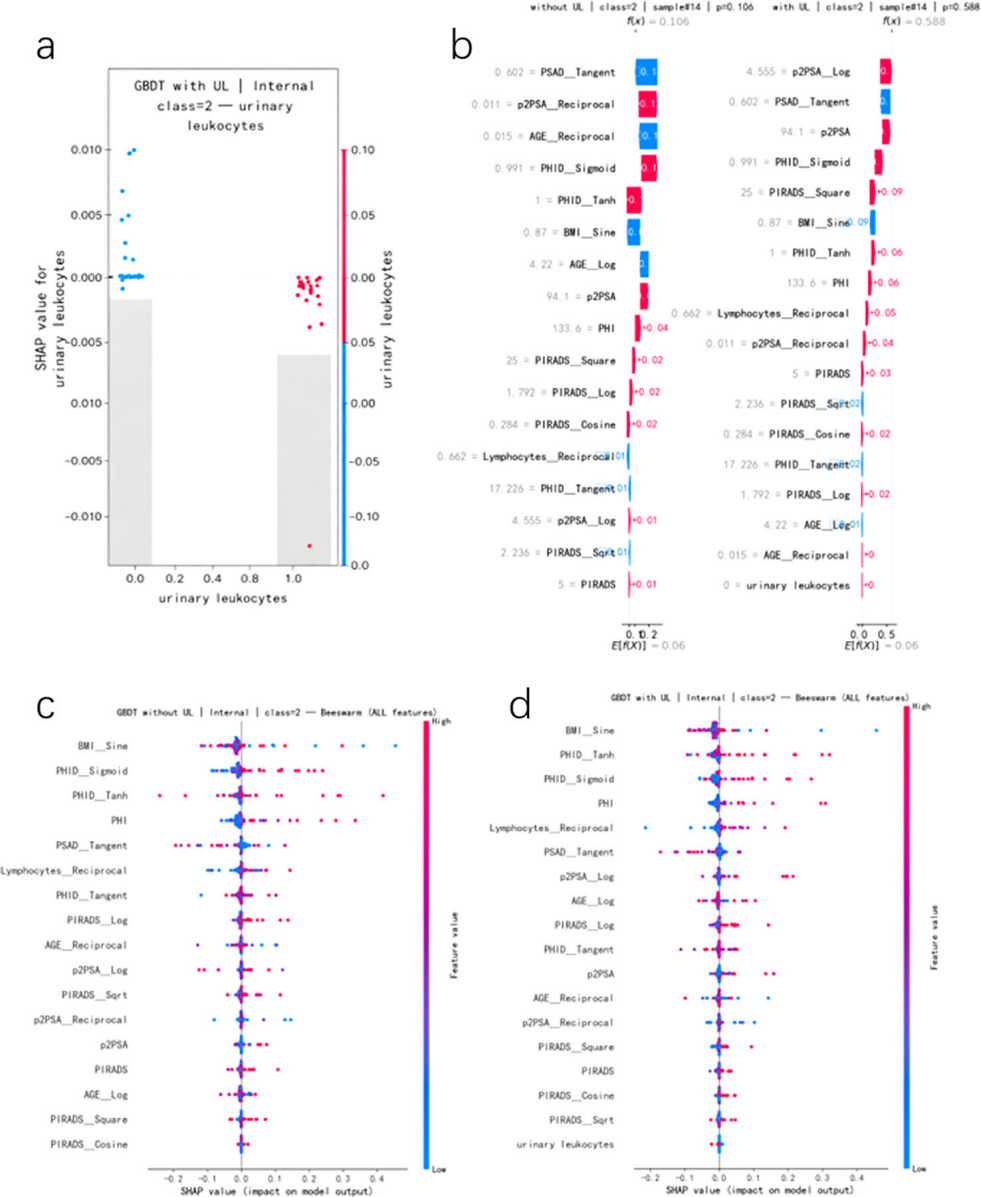
SHAP-based interpretation of the gradient boosting model including urinary leukocytes (UL) for predicting high-risk prostate cancer (class 2). **(a)** Univariate SHAP dependence plot for UL, showing that SHAP values are predominantly mildly negative in UL-positive cases, indicating a down-weighting effect on high-risk predictions. **(b)** Global feature importance ranked by mean absolute SHAP values for class 2 predictions, demonstrating that PSA-related indices and MRI-derived features dominate model output, while UL contributes modest but non-zero information. **(c)** Paired waterfall plots for a representative case (sample #14), comparing the model without UL **(c)** and with UL **(d)**. This example illustrates that the inclusion of UL does not directly increase high-risk probability, but instead reshapes the contribution of other predictors by distinguishing inflammation-related from non-inflammatory PSA elevations, allowing high-risk patterns to be more clearly expressed in UL-negative cases. The x-axes of SHAP dependence plots reflect transformed feature values used for model training rather than raw clinical measurements.

Using the internal test set, we computed SHAP values for the gradient boosting model including urinary leukocytes (UL), focusing on predictions for high-risk prostate cancer (class 2), in order to dissect the contribution of individual features to the model output. Global feature importance ranked by mean absolute SHAP value showed that nonlinearly transformed PSA-related indices and MRI-derived features (e.g. BMI:Sine, PHID:Tanh, PHID:Sigmoid, PHI, and PSAD:Tangent) were the principal drivers of high-risk predictions. In contrast, urinary leukocytes contributed more modestly, ranking in the lower tier of all features but exhibiting a small yet consistent impact (**Figure 6b**). This pattern is consistent with the intended role of UL as an inflammation-related contextual marker designed to complement, rather than replace, conventional quantitative risk indicators.

Univariate SHAP analysis for UL (**Figure 6a**) further clarified the directionality of its effect. Among UL-positive patients (UL = 1), SHAP values for the high-risk class were predominantly mildly negative, indicating that the model systematically down-weights the predicted probability of high-risk prostate cancer when urinary leukocytes are elevated. In contrast, UL-negative patients (UL = 0) exhibited SHAP values clustered around zero, with a subset showing slightly positive contributions. This finding suggests that, in the presence of abnormal PSA or MRI findings, the absence of urinary inflammatory signals is more compatible with a high-risk phenotype. Importantly, this behavior supports the interpretation of UL as a contextual inflammation marker rather than a direct indicator of tumor aggressiveness.

To illustrate this mechanism at the individual level, we examined a representative high-risk case (sample #14) and plotted paired SHAP waterfall diagrams for models trained without and with UL (**Figure 6c**). In the baseline model without UL, the predicted probability for class 2 was approximately 0.11. In the model including UL, although this patient was UL-negative and the direct SHAP contribution of UL itself remained close to zero, the overall feature attribution pattern became more sharply aligned with a high-risk profile. Specifically, the positive contributions of established PSA- and MRI-related features, such as p2PSA:Log, PSAD:Tangent, p2PSA, PHID:Sigmoid, and PIRADS:Square, were more coherently expressed, resulting in an increased predicted probability for class 2 of approximately 0.59. This observation indicates that UL does not increase risk by exerting a large direct effect on individual predictions; rather, its inclusion during model training improves the contextual separation between inflammation-related and non-inflammation-related PSA elevations, allowing canonical high-risk features to express their effects more consistently.

Taken together, the SHAP analyses demonstrate that although urinary leukocytes contribute only modestly in terms of global feature importance, their effect is directionally consistent, clinically interpretable, and mechanistically coherent. By delineating the boundary between inflammatory and non-inflammatory causes of PSA elevation, UL refines probability calibration and reduces misattribution of benign PSA increases to aggressive malignancy. Accordingly, UL should be regarded as an inflammation-related contextual modifier within the risk prediction framework, rather than as an independent tumor biomarker for high-risk prostate cancer.

## 4 Discussions and conclusion

In this bi-center, real-world cohort, we developed a nonlinear stacking ensemble framework for multi-class prostate cancer risk stratification and demonstrated its robust performance across internal and external datasets. By combining predefined nonlinear feature engineering with stability-oriented LASSO selection, the framework achieved strong discrimination and calibration without reliance on advanced imaging pipelines beyond PI-RADS or on costly omics data. The stacking strategy consistently outperformed individual base learners, yielding multi-class AUCs of approximately 0.96 and 0.93 in the internal and external cohorts, respectively, while maintaining satisfactory cross-center generalizability. These results indicate that integrating heterogeneous learners within a calibrated ensemble can effectively capture complex, nonlinear relationships among routinely available clinical and biochemical variables.

Building on this modeling backbone, we systematically evaluated the incremental contribution of urinary leukocytes (UL), a routinely collected but often overlooked urinalysis parameter. Rather than treating UL as an independent tumor biomarker, we assessed its value in terms of calibration, discrimination, and decision-level utility. Across analyses, UL provided modest but consistent improvements in net clinical benefit, particularly in the clinically ambiguous PSA gray zone (4–10 ng/mL), with minimal increase in false-positive classifications. *Post hoc* SHAP analyses conducted on a representative gradient boosting base learner suggested that UL exerts a directionally consistent effect: UL-positive cases tended to receive mildly negative contributions to high-risk predictions, whereas UL-negative cases clustered closer to neutral or slightly positive contributions. Importantly, interpretability analyses were confined to individual base learners and original clinical features, acknowledging that the stacked ensemble itself prioritizes predictive performance over inherent transparency. Within this scope, the observed patterns support a clinically intuitive role of UL as an inflammation-related contextual modifier that refines risk estimation by helping distinguish benign PSA elevations from patterns more compatible with clinically significant disease.

Several limitations warrant consideration. Although data were obtained from two centers, subgroup sample sizes—particularly among UL-positive patients and those with PSA >20 ng/mL—remain limited, which may reduce the stability of subgroup-level estimates. In addition, UL was treated as a semi-quantitative, essentially binary variable derived from routine urinalysis, potentially underrepresenting the full spectrum of urinary inflammatory activity and precluding exploration of dose–response effects. Finally, the retrospective design introduces the possibility of unmeasured confounding and spectrum bias despite external validation. Future studies with larger, prospectively collected cohorts and more granular urinary inflammatory markers are needed to further validate these findings and to clarify how such information can best support biopsy decision-making.

Overall, this work highlights a pragmatic strategy for evaluating the incremental clinical utility of inexpensive, noisy clinical variables within modern machine learning pipelines by explicitly linking feature inclusion to decision-analytic benefit rather than predictive performance alone.

## Data Availability

The raw data supporting the conclusions of this article will be made available by the authors, without undue reservation.

## Supplementary material

Supplementary Figure 1 Distribution of predicted high-risk probabilities in the PSA 4–10 ng/mL gray zone among non-high-risk patients. Histograms show the distribution of predicted probabilities for high-risk prostate cancer (class 2) among patients with PSA levels between 4 and 10 ng/mL who were histologically confirmed as non-high-risk (Gleason score < 8). Results are shown separately for the internal test cohort (left) and the external validation cohort (right). Blue bars represent predictions from the base model without urinary leukocytes (UL), while orange bars represent predictions from the model incorporating UL. The vertical dashed line indicates the prespecified decision threshold (p = 0.5) used for high-risk classification. In both cohorts, the majority of non-high-risk patients exhibit low predicted probabilities well below the decision threshold. Incorporation of UL primarily reshapes the lower-probability distribution rather than inducing additional high-risk classifications. Notably, no non-high-risk patients in either cohort crossed the high-risk threshold after inclusion of UL, indicating that UL does not increase false-positive high-risk predictions in the PSA gray-zone population.

Supplementary Table 1 Features with various nonlinear transformations.
