# Supplementary material for: Role of urinary leukocytes in the risk stratification of prostate cancer using nonlinear stacking learning strategy: a bi-cohort diagnostic study
Source: Front Oncol. 2026 Mar 2;16:1762494. doi: 10.3389/fonc.2026.1762494 (PMC12989820; doi:10.3389/fonc.2026.1762494)
Supplement: Supplementary Figure 1 — Distribution of predicted high-risk probabilities in the PSA 4–10 ng/mL gray zone among non-high-risk patients. Histograms show the distribution of predicted probabilities for high-risk prostate cancer (class 2) among patients with PSA levels between 4 and 10 ng/mL who were histologically confirmed as non-high-risk (Gleason score < 8). Results are shown separately for the internal test cohort (left) and the external validation cohort (right). Blue bars represent predictions from the base model without urinary leukocytes (UL), while orange bars represent predictions from the model incorporating UL. The vertical dashed line indicates the prespecified decision threshold (p = 0.5) used for high-risk classification. In both cohorts, the majority of non-high-risk patients exhibit low predicted probabilities well below the decision threshold. Incorporation of UL primarily reshapes the lower-probability distribution rather than inducing additional high-risk classifications. Notably, no non-high-risk patients in either cohort crossed the high-risk threshold after inclusion of UL, indicating that UL does not increase false-positive high-risk predictions in the PSA gray-zone population. [file DataSheet1.docx]

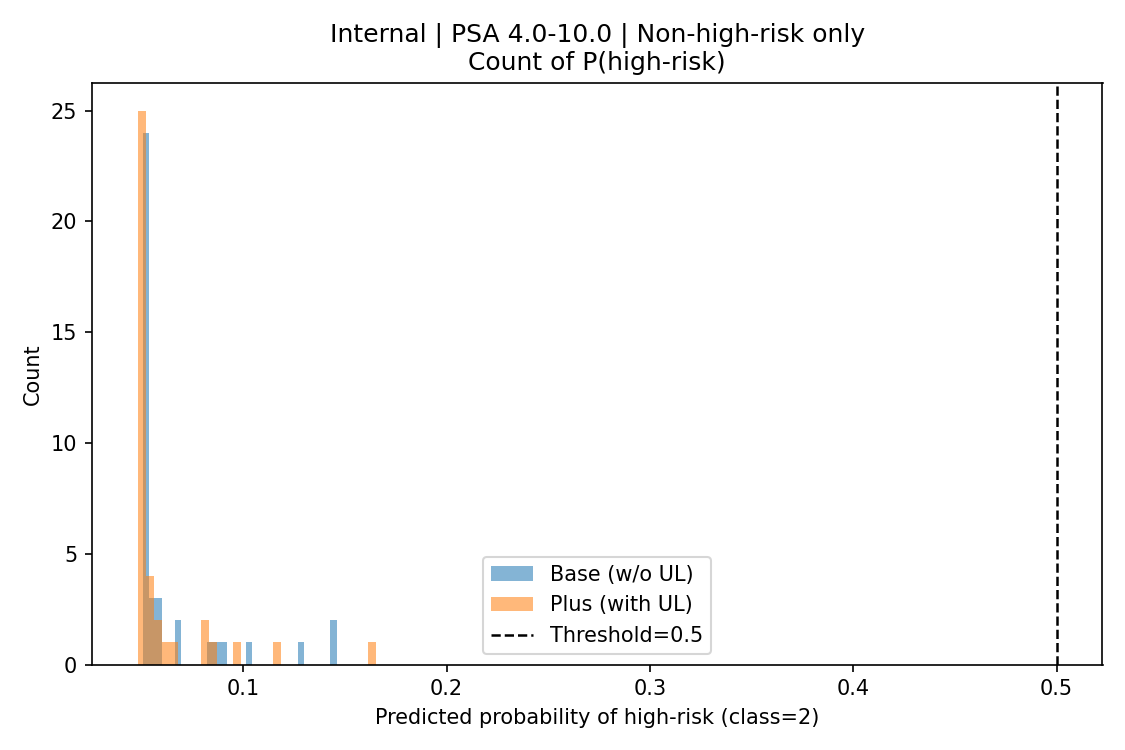

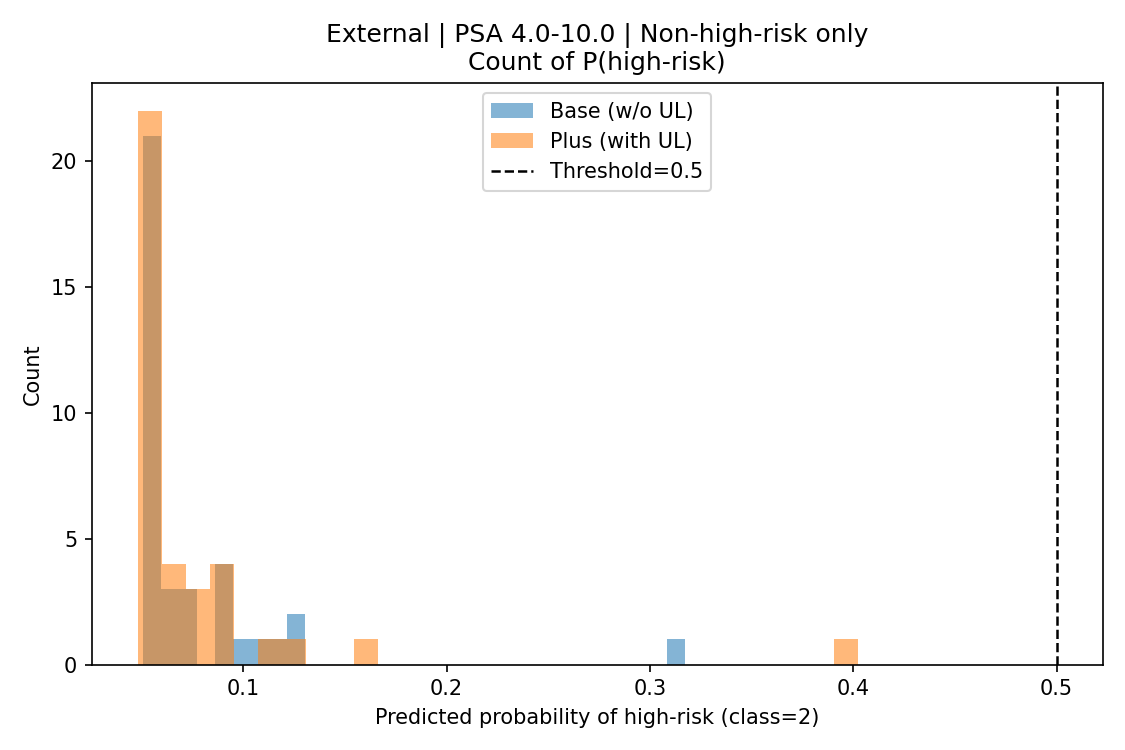


Supplementary Figure 1. Distribution of predicted high-risk probabilities in the PSA 4–10 ng/mL gray zone among non-high-risk patients.

Histograms show the distribution of predicted probabilities for high-risk prostate cancer (class 2) among patients with PSA levels between 4 and 10 ng/mL who were histologically confirmed as non-high-risk (Gleason score < 8). Results are shown separately for the internal test cohort (left) and the external validation cohort (right).

Blue bars represent predictions from the base model without urinary leukocytes (UL), while orange bars represent predictions from the model incorporating UL. The vertical dashed line indicates the prespecified decision threshold (p = 0.5) used for high-risk classification.

In both cohorts, the majority of non-high-risk patients exhibit low predicted probabilities well below the decision threshold. Incorporation of UL primarily reshapes the lower-probability distribution rather than inducing additional high-risk classifications. Notably, no non-high-risk patients in either cohort crossed the high-risk threshold after inclusion of UL, indicating that UL does not increase false-positive high-risk predictions in the PSA gray-zone population.

Supplementary Table 1. Features with various nonlinear transformations

| Feature_name | Original_variable | Transformation | Unit_original |
| --- | --- | --- | --- |
| AGE__Log | AGE | log1p | years |
| AGE__Reciprocal | AGE | reciprocal | years |
| BMI__Sine | BMI | sin | kg/m^^2^ |
| Lymphocytes__Reciprocal | Lymphocytes | reciprocal | ×10^^9^/L |
| PHI | PHI | original | unitless index |
| PHID__Sigmoid | PHID | sigmoid | PHI / mL |
| PHID__Tangent | PHID | tan | PHI / mL |
| PHID__Tanh | PHID | tanh | PHI / mL |
| PIRADS__Cosine | PIRADS | cos | score (1–5) |
| PIRADS__Log | PIRADS | log1p | score (1–5) |
| PIRADS | PIRADS | original | score (1–5) |
| PIRADS__Sqrt | PIRADS | sqrt | score (1–5) |
| PIRADS__Square | PIRADS | square | score (1–5) |
| PSAD__Tangent | PSAD | tan | ng/mL/cm^^3^ |
| p2PSA__Log | p2PSA | log1p | pg/mL |
| p2PSA | p2PSA | original | pg/mL |
| p2PSA__Reciprocal | p2PSA | reciprocal | pg/mL |
